# “Familiar Artifice”: Ways of Telling in the Short Story, Psychoanalysis, and Alice Munro’s “The Moons of Jupiter”

**DOI:** 10.1177/00030651221077312

**Published:** 2022-04-22

**Authors:** Rosemary Rizq

**Keywords:** Alice Munro, fable, identification, narrative coherence, short story, *Totem and Taboo*

## Abstract

Theoretical ideas about “narrative coherence” and “autobiographical competence” remain prevalent in contemporary therapeutic culture, and are frequently deployed in the service of the patient’s producing a narrative “I” that can tell its own story. A preference for novelistic accounts of the self is countered here by proposing the short story form as an alternative model for the telling of a self within psychoanalysis. Alice Munro’s “The Moons of Jupiter,” the roots of the short story form in fable, and a rereading of Freud’s *Totem and Taboo* are used to illuminate how the short story may be seen as an exemplary tale paralleling the origin of the self in its identification with the other.

*Men can do nothing without the make-believe of a beginning.*

—George Eliot (1876, p. 3)

“Even as I most feverishly, desperately practise it,” Alice Munro (1972) writes, “I am a little afraid that the work with words may turn out to be a questionable trick, an evasion (and never more so than when it is most dazzling, apt and striking) an unavoidable lie” (p. 182). Munro’s moral concern with artifice, with the way “work with words” risks turning out to be a “questionable trick,” is something to which we might well pay heed. For these days, of course, everyone has a story. In popular culture there has been an exponential rise in the use of digital and social media, along with self-help groups and other platforms enabling us to assemble, construct, narrate, and defend our particular story: to fashion an identity through which we want to be known. As psychoanalysts, though, we might prefer, like Munro, to remain skeptical of the patient’s—and indeed our own—“work with words”; for the account of a life that is brought to therapy can all too readily be molded into a sequential narrative akin to a novel, whose coherence is incommensurate with the fragmentary, irreconcilable, or enigmatic aspects of experience. Well-rehearsed scripts and chronological plotlines establishing a seamless, self-directed, totalizing account of the self’s past, present, and future not only alert us to the likely false connections woven into this smoothly narrated version of the self; they also signal the potential for an untrustworthy gap to open up between language at its “most dazzling, apt and striking” and the complexity and intensity of the world it aims to describe.

The idea that the telling of the patient’s story in therapy might be akin to the unfolding of a novel is not, of course, new. But we should ask what features of this literary genre might make it a suitable model for the telling of a self. There is a vast literature on the theory of the novel (see, e.g., Bakhtin 1981; Lukacs 1916; Mazzoni 2017; McKeon 2000) and considerable disagreement among literary theorists about how best to define the genre. Indeed, the novel’s long historical evolution and protean form make any attempt at specifying its distinguishing features almost impossible. However, Brooks (1984) reminds us how it has been “the great nineteenth-century narrative tradition” that “conceived certain kinds of knowledge and truth to be inherently narrative, understandable (and expoundable) only by way of sequence, in a temporal unfolding” (p. xii). This interest in time and sequence, tied to the novel’s “central conventions of realism” (May 2012, p. 176), is the feature that has most clearly come to influence the fields of counseling and psychotherapy. It lies embedded in notions of “narrative coherence” (Schafer 1980) and “autobiographical competence” (Holmes 1993) and in the attempt to produce a narrative “I” that can tell its own story: an active quest for reality and identity that is deemed by some (e.g., Roberts 2000) to be the principal aim and function of therapy. Yet if the purpose of psychoanalysis is, as Steedman (1992) suggests, “to give back to the patient the story of his or her life, welded into a chronological sequence and narrative coherence, so that at the end of it all, the coming to psychic health might be seen as the re-appropriation of one’s own life story” (p. 172), then we might want to ask, as does Walsh (2017), whether and to what extent the psychoanalytic subject is able to give any account of him- or herself that does not recruit the linear, sequential mode of narrative typical of the novel.

By way of interrogating these novelistic forms of discourse in some currently popular models of therapy, I want to propose a possible alternative literary form that Freud himself suggests in his early case histories. Freud (1895) was notoriously dismayed by the way these appeared to read more like short stories than scientific treatises. “I must console myself,” he writes in his case study of Elisabeth von R., “with the reflection that the nature of the subject is evidently responsible for this, rather than any preference of my own” (p. 160). Freud’s own “work with words” here is already mobilizing the “questionable trick” of which Munro speaks; for the idea of something in “the nature of the subject” leaves us wondering whether Freud is referring to the subject in terms of the field, the discipline of psychoanalysis, or whether he is hinting at the psychoanalytic subject in terms of the self and its constitution. I take seriously the latter idea: that there is something in the nature of the self and how it is brought into being that might require something closer to the form of a short story for its articulation than the narrative modes of telling so often favored in contemporary therapeutic culture.

Why might this be so? The relationship between psychoanalysis and literature has traditionally been seen by some (e.g., Derrida 1975; Felman 1977; Kirova 1997) as a troubled one, as I have discussed elsewhere (Rizq 2018). Moving on from these arguments, however, I want to start by thinking of narrative as a way of presenting reality to the mind: of stories as a kind of epistemological category (Jameson 1989). Of interest here are the literary methods of the short story, and the means by which the reader is oriented to a particular kind of experience and knowledge of the world and the self. It is beyond my scope here to detail in full the range and variety of ways in which this is said to be different from the kind of knowledge and experience that is conveyed by the novel. But we might remember that the realist novel, especially the *Bildungsroman*, aims to generate an illusion of life by revealing actions and everyday details of the social, psychological, and material world that confer a sense of authenticity and verisimilitude. It aims to offer a coherent, well-plotted narrative with an emphasis on resolution of conflict. Modernist novels have, of course, significantly complicated these features, aiming to “illumine the mind within” as Virginia Woolf (1929) argues, “rather than the world without” (p. 121) by creating an illusion of mental life via an intense focus on the minute details of a shifting inner consciousness and the vagaries of personal subjectivity. And while postmodernism has since considerably problematized and extended its formal possibilities, the novel’s overriding concern with fullness, plenitude, and an extensive view of the world—including the inner world—could be said to contrast with the short story’s focus on the momentary, the singular, and the revelatory.

The short story can be understood as a form that is “compressed, unified and plotted” (Patea 2012, p. 3), rendering perception “in a mode close to the way in which we experience and know the world: occasionally, in fragments” (p. 19). By virtue of its brevity and concision, it is capable of generating for the reader a kind of experience quite different from that engendered by the novel. It rests on something closer to a metaphysical ontology, created by a focus not on the temporality and linearity of everyday life but rather on what Baxter (2008) calls the “widened moment” that refuses to recast “the incommensurate into an arbitrary continuity” (Trussler 1996, p. 562). The novel, particularly the “standard novel of plotted suspense” (Rader 1993, p. 80 n. 6), commonly recruits an explanatory epistemology and proceeds by way of specifying the contextual details that render the social world recognizable and familiar. The short story, by contrast, tends to recruit a revelatory epistemology, proceeding via a “logic of unveiling” (Baxter 2008) that generates a moment of secular ephiphany or insight. Novels are frequently discursive, aiming to expand or embroider on a particular theme, while a short story is capable of condensing or distilling experience. It is likely to focus on a single event, emotion, or situation, aiming to capture the essence of a feeling or experience. It conveys, as Poe (1846) suggests, a “unity of impression” or a single, self-contained effect. Finally, from an historical perspective, the novel stems from the oral tradition of the saga and the epic: lengthy, complex forms rooted in classical stories of travel, warfare, and heroic deeds. The short story, by contrast, is a briefer narrative form deriving from the ancient traditions of myth, biblical verse, parable, fable, and romance. It is bound up with the experience of the sacred. Indeed, in its tendency to engage with what Rohrberger (2004) calls “a mystical world of paradox and ambiguity, of shadows and shifting perspectives governed not by rational order but by intuition and dream logic” (p. 6), we might say that the short story constitutes “not a form of knowledge, but a challenge to knowledge” (Leitch 1989, p. 133).

While there is a growing academic literature on the short story (see, e.g., Lounsberry et al. 1998; May 1996, 2013; O’Connor 1963; Rohrberger 1998), I do not intend, nor is it within my area of expertise, to elaborate on the theory of the genre. Rather, I want to borrow from literary criticism to focus more narrowly on what it is about the short story’s particular “way of telling” that might shed light on the account of a life produced within a psychoanalysis. I will use the example of Munro’s *The Moons of Jupiter* (1983), a collection of short stories in which she offers a sequence of eleven tales plotting the lives and experiences of women at different stages of their lives. The content of each story varies enormously, but all revolve around her protagonists’ shared experiences of work, relationships, and family life, as well as their confrontation with aging and death. The final, title story of the collection is justly celebrated not only by Munro’s reading public but also by literary critics including Howells (1997), who suggests that “The Moons of Jupiter” is “arguably the most significant turning point in Munro’s fiction-writing career” (p. 67). Mayberry (2009) calls it “one of Munro’s most intensely focused examinations of the capabilities and limitations of narrative” (p. 30). While these writers and a number of other critics, notably Carrington (1989), Heble (1994), McIntyre (2009), and Redekop (1992), provide stimulating close readings of the story, none of them sets out specifically to examine its implications for a psychoanalytic understanding of the self and its constitution. I am aware, of course, that this territory includes debates and theoretical ideas that are likely to be extremely familiar to psychoanalysts and literary critics alike; but I hope to approach the topic as freshly as possible via an exploration of the kind of subjectivity Munro describes and dramatizes in her particular way of telling in the story. Indeed, my purpose here is not to offer a psychoanalytic reading of Munro’s tale, thereby perpetuating the privileged theoretical position often assumed by psychoanalysis over literature—what Felman (1977) has called a “relationship of subordination” (p. 5)—so much as to find new ways of illuminating psychoanalytic ideas through my reading of her story. Fittingly, I will remain close to Munro’s own logic, which consistently focuses on the limits inherent in language and representation. Lacanian metapsychology, with its problematic of the real, offers an important touchstone here, as does Keenan’s philosophy of reading, which interrogates the fable in order to explore how subjectivity is installed in the reader via a process of substitution and identification. I will go on to offer a rereading of Freud’s *Totem and Taboo* (1912–1913), where I follow Lacan in taking the view that the self finds itself in the image of the other. I suggest that, like “The Moons of Jupiter,” *Totem and Taboo* may be seen as an exemplary tale laying bare the fictive origins of the self. I will conclude that the fabular lineage of the short story form makes it a particularly suitable literary model for the constitution of the self within psychoanalysis.

## “The Moons of Jupiter”

In the opening pages of “The Moons of Jupiter,” we find a middle-aged, divorced Janet settling her elderly father into the cardiac unit of a Toronto hospital. He is wired up to an EKG: “On the screen,” says Janet, “a bright jagged line was continually being written. The writing was accompanied by nervous electronic beeping. The behaviour of his heart was on display. I tried to ignore it. It seemed to me that paying such close attention—in fact, dramatizing what ought to be a most secret activity—was asking for trouble” (pp. 217–218).

The alert reader will be aware, even at this early point in Munro’s tale, that the “jagged line” that is “continually being written” here is not simply an erratic heartbeat. It points a metafictional finger, so to speak, at the act of writing itself: the art of representing the heart’s “most secret activity,” something that is usually taken to be invisible. But what is it about “paying such close attention” to writing that leads to trouble? Munro’s carefully choreographed and extraordinarily stylized story of only a few pages illuminates how the artifice of language inevitably distances us from reality; and how narrative design and order, as well as the act of naming in the story, rely on the mimetic power of language to evoke mystery, pathos, and an elegiac sense of loss in the reader while at the same time performing its ineluctable failure to adequately represent the world, the self, and the other.

Janet is coming to terms with the impending death of her father, as well as the refusal of her eldest adult daughter to communicate with her. The story, told over seven short sections that loop back and forth over the space of a few days, is not only of how she makes the complex inner psychological adjustments necessary to confront the task of mourning and letting go within her family relationships; it is a tale that dramatizes how Janet achieves moments of a new self-awareness. It seems unlikely then that Munro’s decision to arrange her material in seven, nonsequential sections is arbitrary; for like the biblical span of seven days that God takes to create the world, Janet herself has limited time before her father’s operation to create a new emotional world. She will do so by restoring a lost sense of connection and understanding with him, as well as with her eldest daughter. The story’s lack of narrative linearity—Janet’s four hospital visits, an overnight stay at her youngest daughter’s apartment, a visit to the planetarium, and a tranquil moment of rest in a Chinese garden—vividly conveys the way Janet’s consciousness and memory orbit around her family and loved ones. A muddled chronology, defying easy summary, not only mirrors the muddle and disorder of life; it mimes the way in which Janet’s memory works retroactively to rework and revise her understanding of her relationships, as well as of herself.

Janet herself is a writer, and the reader is given to understand that she will struggle with the versions of other people she has created in her mind. We see how this makes it difficult for Janet to face and cope with her father’s manifest vulnerability and to think about him as a person with an independent life and history of his own. She knows that as a young boy he ran away from home in search of a better life, but she hadn’t cared “to think of his younger selves,” she admits: “Even his bare torso, thick and white . . . was a danger to me; it looked so strong and young. The wrinkled neck, the age-freckled hands and arms, the narrow, courteous head, with its thin gray hair and mustache, were more what I was used to” (p. 220). It is clear then that Janet’s relationship with her father is predicated on a story she has fabricated around him: “his independence, his self-sufficiency, his forbearance” (p. 220). When she tells people about him, she insists: “He worked in a factory, he worked in his garden, he read history books. . . . he never made a fuss” (p. 220).

But following the news that her eldest daughter, Nichola, wants to be “incommunicado for a while” (p. 221), Janet begins to examine herself afresh. Wryly recalling her younger self with her own friends, she thinks, “How thoroughly we dealt with our fathers and mothers, deplored their marriages, their mistaken ambitions or fear of ambition, how competently we filed them away, defined them beyond any possibility of change. What presumption” (p. 222). Indeed, Janet now starts to realize that what she believes about Nichola—that she is “sly and solitary, cold, seductive” (p. 223)—is likely to be contradicted by people who know otherwise. On returning to the hospital, she begins to question her understanding of her father, recognizing that the version she has always held of him is partial, colored by her own perspective, and distorted by self-interest. It was not true that “he never spoke regretfully about his life,” she realizes. “It was just that I didn’t listen” (p. 225).

But in the face of an imminent heart operation, her father is reworking his own version of himself as part of his preparations for death. A further fiction is invoked as he quotes the line “Shoreless seas,” from “Columbus,” a poem by Joaquin Miller that he is trying to recall. Like the jagged lifeline of the EKG that illuminates the heart’s vital, yet hidden biological rhythms, poetry here illuminates the heart’s essential yet concealed psychological activity as it prepares for its final journey toward death and the New World of the unknown. Yet skepticism about the capacity of poetry or any fiction to console in the face of mortality remains foregrounded: “If there’s anything you can’t explain right away, there’s a great temptation to—well, to make a mystery out of it” (p. 226), he remarks; and Janet, “feeling an appalling rush of love and recognition” (p. 226), believes her father is referring to the soul. But both maintain a certain wariness about the false comforts of religion that mean “playing tricks on yourself” (p. 226). Inasmuch as Janet’s father wants to believe in the stories he has been reading in magazines that tell of people’s comforting near-death experiences, he maintains a level of suspicion, if not outright cynicism, when faced with any attempt to make death meaningful, comprehensible, or bearable: “It’s all in whether you want to believe that kind of thing or not” (p. 226).

Janet’s visit to the planetarium, ostensibly to distract herself from her anxiety about her father’s impending operation, unmistakably signals the central event of the story. As she sits down with an audience of noisy schoolchildren, a theatrical presentation announced by “some splendid, commanding music” (p. 230) turns the bowl of the planetarium’s ceiling into dark blue night sky, “an orchestrated image” according to Howells (1998) “of infinite space inside a closed dome” (p. 83). It is at once a mythic vision of the cosmos, dramatizing the shifting patterns of a universe whose distant meaning lies beyond any human understanding, as well as an image of the patterning of meaning that constitutes the narrative text. The immensity and significance of the transcendent vision is set alongside the apparent banality and meaninglessness of everyday life, indexed here by the audience of indifferent children “crackling their potato-chip bags” (p. 230). At first it is the illusion of reality that is foregrounded—“the stars came out not all at once but one after the other the way stars really do come out at night, though more quickly” (p. 230)—and the “stunning facts” are confidently listed to convey the “innumerable variations, innumerable repetitions” (p. 231) of the galaxies within the universe. Subsequently, though, we are told “realism was abandoned for more familiar artifice” (p. 231); a model or map of the solar system is invoked to convey in more “elegant style” the movement of the planets within the vast cosmos. Indeed we, like the audience watching the show, are being offered different ways of representing and dramatizing the unfathomable, unknowable, and mysterious workings of the universe and our place within it. A sense of the radical insufficiency of any knowledge obtained by these forms of representation is, however, always present. Janet learns that Mercury now rotates three times as it circles around the sun, instead of just once as was previously understood. These updated facts about planetary activity mirror on a cosmic scale the way in which Janet is in the process of updating the meaning and significance of events and relationships in her life. Knowledge can only ever be provisional: “Why did they give out such confident information, only to announce later that it was quite wrong?” (p. 231). Janet’s skepticism here is not only about the validity of scientific knowledge; it registers Munro’s profound doubts about the capacity of language to model the world and to frame life’s excess and complexity in all its “innumerable variations, innumerable repetitions.” Perhaps it is not surprising then that at the end of the show Janet remains unimpressed by the artifice behind the presentation: “the music, the church-like solemnity, simulating the awe that they supposed they ought to feel” (pp. 231–232). The cosmic drama she has watched fails to do justice to the “horrible immensities” (p. 231) of the universe; and like the children who do not comment on what they have seen but are more interested in “eatables and further entertainment” (p. 231) she refuses the comforting vision of transcendence on offer, much as her father has earlier refused the false comfort offered by the magazine stories of near-death experiences. Awe, she feels, is something to be felt only in the face of the real: “once you know what it was, you wouldn’t be courting it” (p. 232).

Returning to the hospital for her father’s last night before the operation, Janet says that she has been to an “exciting” show at the planetarium, telling him “it’s like a slightly phony temple” (p. 232). She immediately regrets her comment: “I had meant that to be truthful, but it sounded slick and superior” (p. 232). The limitations of words to convey truth can be shaming, Munro hints, not only because they utterly fail to convey the reality of experience and mortality, but because, like the word “exciting,” they can also convey something false: something closer to a caricature than the truth. Ashamed of her “slick and superior” language, Janet makes a final effort to forge an emotional connection with her father, asking him to list the moons of Jupiter, and they engage in a mutual attempt to remember and name each one: Io, Europa, Ganymede, Callisto. Naming here, for Munro, is much more than simply providing a label; for here the process of naming the moons testifies to the power of fiction to create and shape the universe into a satisfying story. The Greek characters after whom the moons are named are rooted in mythology, a classical narrative used to understand and manage the mysteries of the universe. It is this mythical narrative that is recruited by Janet and her father to create an illusion of comfort before their inevitable parting.

The story’s seventh and final section concludes with a flashback to Janet’s brief sojourn in a Chinese garden after leaving the planetarium the previous day. As Janet sits peacefully on her own, the rearranged chronology of the story allows Munro to hint at the biblical day of rest following God’s creation of the world. Like the story itself, the garden is a fiction, a representation of something that belongs to another time and place; but its “familiar artifice,” like the planetarium’s, seems to calm and soothe Janet before she returns to the hospital for the final meeting with her father. In the distance, she sees “a girl who reminded [her] of Nichola” (p. 233) and realizes that just as she will have to let her father go into his operation, so too she will have to let Nichola go into adult life, independent of her mother: “She was one of the grown-up people in the world now” (p. 233).

## Forms of Stories

“I don’t really understand a novel,” says Munro (quoted in Rothstein 1986). “I don’t understand where the excitement is supposed to come in a novel, and I do in a story. There’s a kind of tension that if I’m getting a story right I can feel right away, and I don’t feel that when I try to write a novel. I kind of want a moment that’s explosive, and I want everything gathered into that” (p. 17).

If, as May (1984) argues, ”the short story is short precisely because of the kind of experience or reality embodied in it” (p. 328), then perhaps it is by way of the “explosive” moment that we can best grasp the genre’s most fundamental way of telling. For unlike the novel, which is propelled by its relationship to what E. M. Forster (1927) calls “the naked worm of time,” a narrative of events or experiences generally arranged in linear sequence, the short story is driven by its relationship to the epiphanic moment: a moment that, by virtue of being extracted from the flow of time, comes to be imbued with particular significance and power. Indeed, as a literary mode that remains close to fable and folklore, as May (1984) reminds us, the short story is acutely sensitive to the manifestation of what Cassirer (1946) refers to as the “momentary deity”: a primitive mode of experience constituted by a “fleeting, emerging, and vanishing mental content” piercing everyday life with an experience of the uncanny or sacred. “In stark uniqueness and singleness it confronts us,” writes Cassirer, “not as part of some force which may manifest itself here, there, and everywhere, in various places and times, and for different persons, but as something that exists only here and now, in one indivisible moment of experience, and for only one subject whom it overwhelms and holds in thrall” (p. 18).

Gordimer (1976) describes the short story’s attempt to depict this ephemeral, phenomenological quality of experience as akin to seeing by “the flash of fireflies, in and out, now here, now there, in darkness” (p. 264). It is a metaphor that vividly evokes the simultaneous, transient glimpses of the universe that Munro describes in the planetarium scene: “the way the stars came out not all at once but one after the other the way stars really do come out at night, though more quickly.” Munro captures this momentary, dissolving perception of what lies beneath life’s surface appearances by embedding it not within a chronologically arranged sequence of episodes over time, but within a more paratactic arrangement: a loose cohesion of incidents that prohibits any easy recourse to presumed connections, reasons, or motives. The story’s seven separate sections index various events taking place over a few days in Janet’s life. Juxtaposed rather than explicitly connected, they are related in a way never unambiguously thematized, allowing Munro to simply point to events rather than attempting to explain their relationship to each other. Indeed, the idea that we might be able to establish a causal connection between contingent events, that we might be able to mold the direction of our lives into a comforting explanatory narrative, is precisely the kind of self-deceiving fiction that Munro’s story seeks both to dramatize and unsettle.

The elimination of causal connections alongside the story’s tightly knit unity of form ensures that the reader must work hard to establish the possible interrelationships between disparate elements in the tale, as well as to grasp what is hinted at or obscured beneath the surface of the text. It is not until the planetarium scene that the reader is presented with what we might think of as the central *mise en abyme*: a scaled-down mirror version of the wider universe that is also a version of Munro’s fictive world. It confronts the reader with an explosively dense metafictional moment in which Munro finally lays bare the artifice that underlies our experience of the world and its “horrible immensities.” It gestures toward the radical insufficiency of language even while it exploits it as a medium that comforts us with “stunning facts” or traditional narratives seeking to reassure and explain. The patterns of the stars, the constellations that Janet sees in the bowl of the planetarium, the scientific models by which we understand the universe are only human constructions imposed on a vast, enigmatic, and ineffable universe. “Constellations do not exist,” writes Milner (2016), “there only exist the stars that compose them” (p. 31). Like Janet, we look up at the starry sky and persuade ourselves that we can see meaningful patterns emerging from the formless cosmic flux above. The Great Bear and Little Bear that Janet mentions to her father in the hospital are created entirely within the imagination; yet somehow they appear to us still, radiantly shining out from the night sky. We navigate the external universe by weaving the chaos of the stars into meaningful patterns, forms, and names, just as we navigate the internal subjective world by constructing patterns of meaning out of the fragments of our experience, from the disparate events and contingencies of our lives. Indeed, when Janet and her father work together in their final meeting to name the moons of Jupiter, their joint effort brings, quite literally, the “momentary deity” into existence. The names of Io, Europa, Ganymede, and Callisto of course derive from classical mythology, itself a series of fictional narratives intended to render the vast intricacies of the universe explicable to the ancient Greeks. Munro seems here to be calling on the short story’s earliest traditions to underline the way naming, giving words to things, itself constructs how we perceive, pattern and understand the world. Yet it is in her very attempt to narrate experience, to “dramatize what ought to be a most secret activity,” that Munro performs the “questionable trick” of writing. By drawing attention to her story as a work of art, as well as to the role of the writer in fashioning or fictioning the world, Munro aims to interrogate, disrupt, and unsettle our ideas about our relationship to reality.

## The Short Story as Fable

Perhaps these notions of fashioning and fictioning bring us closer to the roots of the short story in fable. The etymology of the word *fable* derives from the Latin *fari*, to speak, as well as the Greek *pharein*, to say. From *fari* we derive fate—that which is spoken—and *fabula*, an account or story, something that is fabricated. To speak, it seems, is always to tell a tale; yet, as Voltaire (1747) comments in the dedicatory letter to *Zadig*, a fable is an “ouvrage qui dit plus qu’il ne semble dire” (p. 22)—a work that says more than it seems to say. Indeed, in the past, fables were often taken to be mythologies, animistic folktales requiring interpretation to uncover what Smith (1915) calls “some general truth pertinent solely to man” (p. 524). While not all short stories are fables, it is certainly true that fables themselves are generally brief, sometimes extremely so; and so we might say, in Gelley’s words (1995), that the fable is a short tale that aims to articulate “narrative examples and moral meanings” (p. 122). It provides a model of ethical conduct not via moral instruction or didactic teaching but rather via instances or comparisons. Something is brought to the reader’s awareness by way of a likeness, an Aristotelian illustration or *paradeigma* inductively pointing to further examples of a more general theme or category. Examples function, suggests Aristotle, “by moving from particular to particular, passing through the universal,” offering “a particular and practical conclusion that is an indication for concrete acting” (quoted in Natali 1989, p. 147). If we stand back for a moment to position “The Moons of Jupiter” in the context of the ten other stories in Munro’s collection, we can see that the entire book revolves around a series of iterative examples comprising the “innumerable variations” and “innumerable repetitions” of women’s lives.

However, Ambuel (2007) reminds us that we need to account for a certain philosophical ambiguity in the notion of a *paradeigma;* for the term refers to a particular instance or example, as well as to the transcendental ideal on which all other instances or examples model themselves. “A *paradeigma* might be an architect’s or sculptor’s model, an image (*eikōn*) of what is to be made,” he writes, “but it can also be an exemplar, the standard against which other things are measured” (p. 8). But what might this mean in Munro’s tale? For it is not as if she is asking us to accept “The Moons of Jupiter” as an example of how to behave; or that Janet is being held up as a moral ideal for us to emulate. Rather, hidden within her story lies the deeper question about language and its relation to the reality we inhabit. To what extent, and with what effect, Munro asks, can the fiction that is language stand as an example of the world it describes and illuminates? Can it say anything truthful, or does its deployment of “dazzling, apt and striking” words merely mislead us? Munro’s skepticism comes to the fore in the central exemplum of the planetarium where, by juxtaposing Janet’s world of relationships alongside the sidereal world of the cosmos, she foregrounds the imaginative partnership entailed by our contact with the world. Like Janet, we are confronted by “familiar artifice,” the fictions by which we understand the “horrible immensities” of the universe. But are these “ordinary “illusions” (Seligman 2018, p. 267) merely a kind of ornamental cover used to stretch over the reality of the world, like the ceiling of the planetarium? Or is there something about the world itself that demands this covering, this fiction, in order to display itself at all? Pushing at these questions a little further, we can see it is not that fiction offers us a limited version of reality, a replica that convincingly simulates the world while unfortunately lacking authenticity in this or that respect; or that it enables us to find ways of modeling things that already exist in the world. The metaphysical opposition between reality and fiction is not to be resolved by recourse to a fictional model purporting to represent the reality of the world. Instead, Munro seems to suggest, it is through the act of writing, of inventing a story, that we are able to fashion or fiction a world for ourselves; that we can bring the world itself into being. Yet even as Munro undertakes this creative act, she remains deeply mistrustful of its authority. She deploys “artifice” as a means of fictioning a world whose claim to a relationship of verisimilitude remains continually open to doubt. Indeed, in ensuring that “realism [gives] way to familiar artifice,” Munro fashions a world whose invented origins, as we shall see, embody and instate a truth about the fiction that inaugurates the self.

## The Short Story as Fable of the Subject

Fables of course, are principally allegories: brief stories used as models of ethical action. “The fable,” writes Keenan (1997), “is offered for example, but for the kind of example that asks to happen in an act of something like imitation or identification, in the rhetorical event of a comparison” (p. 46). What does this mean, and how might the notion of the fable as exemplary tale help us understand the relationship between the short story and the psychoanalytic concept of the subject? In his theory of reading, Keenan draws on the Aesopian fable of the raven who pretends to be an eagle to illuminate the way identification with the other is the borrowed basis of the self. Starting off without a name of his own, the raven decides to call himself an eagle, hoping to catch a lamb in his claws like a bird of prey. Captured by a shepherd after failing in his hubristic task, the raven is asked what kind of bird he is. He responds by retelling the fable and calling himself “raven” for the first time, thus restoring to himself a name he never knew he had. “The raven speaks,” writes Keenan, “and finally says, ‘I am,’ starting from a feint, a fiction, a borrowed figure” (p. 67). The reader of the fable, he suggests, is similarly asked to identify with the story’s protagonist: to compare him- or herself with the raven and “to assume its name and follow its example, in order to learn not to compare yourself with what you’re not” (p. 66). Like the raven, who has made the error of substituting himself for another, then, the reader is recruited by the fable’s address into miming the very rhetorical mechanism of substitution by which the protagonist comes into being as a subject. It is an error, suggests Keenan, that consigns subjectivity to a constitutive alterity of which it will always remain unconscious.

Keenan’s interest lies in the way reading restores subjectivity, installing a certain responsibility in the face of the undecidable. But it is what he has to say about the fabular basis of the self that I think most helps us freshly approach the issues of self-formation raised by Munro’s story. I want to return to Freud here, for in the final section of *Totem and Taboo* (1912–1913) he was once again to write a short story: not a case study this time, but rather a story about the anthropological origins of the oedipus complex and the foundation of society, religion, and culture. In his tale, Freud proposes a decisive, historical event in primitive times: the existence of a tyrannical, primal Father and the tribal horde of sons who kill and devour him. This “memorable and criminal deed,” Freud suggests, marked the beginning of religious principles and moral restrictions, as feelings of guilt and remorse in the band of brothers led eventually to the creation of taboos. In my rereading of Freud, I am mindful of Lacan’s view that “the important feature of *Totem and Taboo* is that it is a myth” (1959–1960, p. 176); indeed, as he writes elsewhere, “the myth is more like a fable” (p. 115 n. 7). Like Aesop’s fable of the raven and the eagle, then, *Totem and Taboo* can be read as a fiction that has something to tell us about the constitution of the self, about how we come to consciousness of ourselves as subjects through identification with the other.

While Strachey (1955) tells us that Freud regarded *Totem and Taboo* as his best-written work, it is clear that Freud himself had considerable doubts about its publication. Indeed, his anxiety can be inferred from a short footnote in the paper: “The lack of precision in what I have written in the text above, its abbreviation of the time factor and its compression of the whole subject matter, may be attributed to the reserve necessitated by the nature of the topic” (p. 141). Perhaps it is worth pausing here; for Freud’s choice of words, his reservation about “the nature of the topic,” is strangely reminiscent of the words used in his case study of Elisabeth von R. some eighteen years earlier. We might recall that in that work Freud expresses concern about how there is something “in the nature of the subject” that means his case histories are likely to be read as short stories rather than scientific reports. In *Totem and Taboo*, Freud’s worry resurfaces again in his references to the “abbreviation of time” and “compression of the whole subject matter,” characteristics that, as we have seen, may be considered to be the sine qua non of the short story, yet which Freud feels—with some dismay perhaps—lend “a monstrous air” (p. 141) to his hypothesis.

In his daring tale of sacrificial parricide, Freud suggests that the tribal brothers unite over their hatred and ambivalence toward the authoritative father of prehistory. “The violent, primal father,” he writes (1913), “had doubtless been the feared and envied model of each one of the company of brothers; and in the act of devouring him they accomplished their identification with him, and each one of them acquired a portion of his strength” (p. 142). Killing the father, Freud seems to suggest, takes place neither out of the sons’ wish for revenge nor out of jealousy of his sexual privilege, but rather in order to acquire his power and identity for themselves. In this way, the eating of the primal father, the totem meal that incorporates the other within the self, implies the annihilation of the other who is now the constitutive foundation of the self. “I the ego am born by assimilating the other, by devouring him, incorporating him,” writes Borch-Jacobsen (1993) in his provocative discussion of *Totem and Taboo.* “Everything . . . begins with murderous, blind identification, all the blinder because there is still no ego to see anything or represent anything to itself at all” (p. 33). Reading *Totem and Taboo* in this dark vein reverses the notion of identification Freud (1917) was subsequently to propose in “Mourning and Melancholia,” where identification is seen as a form of desire (“identification is a preliminary stage of object-choice” [p. 249]). Here desire is instead understood as identification and we are given an example of the identity of an ego that cannot preexist the event of imitation, of identification; it is as if the very essence of subjectivity lies not within the self, but within the other who is desired as model: “the imitant,” says Lacoue-Labarthe (1990), “has to be nothing in and of itself or must . . . have ‘nothing characteristic of itself’. I must not therefore already be a subject” (p. 82). It seems then we can only come into being, we can only be created, through identification. It is a borrowing of that which will never be returned, a ruthless plagiarizing of the other through which we incorporate an identity whose alterity we subsequently repress or eliminate. Through this act of identification the desiring subject, the one who longs to be a subject as the other is a subject, is inaugurated.

But what can it be “in the nature of the topic” that makes Freud protest further on in his footnote that “it would be as foolish to aim at exactitude in such questions as it would be unfair to insist on certainty” (p. 141)? Along with Lacan (2008), who comments, ironically, that “we have seen orangutans. But not the slightest trace has ever been seen of the father of the horde” (p. 113). I would like to suggest that whatever else it is, Freud’s wonderfully speculative story is an exemplary tale; indeed, it is one that Freud (1921) himself was to call a “Just-so Story” (p. 122). It mimes the inexactitude, the undecidability of its own fabulous origins. We might remember that Freud at first seems to argue that the putative historical accuracy of the events he describes is irrelevant: the reality might never have taken place at all. The sons’ remorse could have been provoked as much by the fantasy of killing the father as by actually killing him in reality: “the mere existence of a wishful *phantasy* of killing and devouring him,” he writes, “would have been enough to produce the moral reaction that created totemism and taboo. . . . psychical reality would be strong enough to bear the weight of these consequences” (p. 160). But Freud’s concern about the “lack of precision” in his hypothesis only serves to make him all the more determined to make a case for the decisive priority of the actual event’s occurrence in reality. In making a comparison with modern-day neurotics, he argues, “neurotics are above all *inhibited* in their actions: with them the thought is the complete substitute for the deed. Primitive men, on the other hand,” he writes, “are *uninhibited*: thought passes directly into action” (p. 161). For primitive men, then, the deed is preeminent. It takes priority over something that in the neurotic will remain at the level of psychic fantasy. And so it is the originary act, the primal murder, which appears to constitute the basis of Freud’s triumphant conclusion: “in the beginning was the Deed” (p. 161).

But in another apparently minor footnote, almost an *esprit de l’escalier* at the end of the essay, Freud enigmatically refers this final phrase back to a scene in Goethe’s *Faust.* It is the moment in which Faust translates the first few lines of Genesis (“In the beginning was the Word”) and, dissatisfied, finds himself substituting “deed” for “Word” in precisely the gesture that Freud himself has just plagiarized. In other words, Freud’s claim to verisimilitude appears to lean not on the authenticity of a potentially verifiable historical event, but rather on a borrowed fictional text, on another Word. Like Faust, Freud is attempting to substitute an act for what is, in fact, a fiction. But if the primal murder, the Deed, is seen as merely a substitute for what will later become, in neurotics, the fantasy of killing the father, we might say that the authority of Freud’s tale rests on the undecidability of a prior fiction, on the generation of a primal fantasy now deemed to be the precondition for an invented beginning. Following Keenan then, it seems the eagle that is *Totem and Taboo* turns out to be a raven after all. It is an exemplary tale, a fable in which Freud stages the very act of identification, of substitution, that itself inaugurates his story of the founding father, which is to say it is a fable about the origin of fable itself. By telling us a fictive story of origins folded within another founding tale, Freud lays bare “the origin of so many things—of social organization, of moral restrictions and of religion’ (p. 142), exposing them to the artifice of their beginnings.

We are beginning to approach more nearly here how the short story’s fabular lineage makes it a genre particularly suited to dramatizing the project of self-knowledge and its limits. Like most of Munro’s characters, Janet is a writer and storyteller whose memory appears to be the principal source and repository of a growing self-awareness. But Munro alerts the reader to the way in which Janet’s memory has formed the basis of a self whose origins lie in a necessary fiction: necessary not just because of some token admission of our limited capacity to know and understand the self, but because of Munro’s awareness of the ineluctable limits to self-possession. When Janet reviews the narrative of her life, she becomes aware, as Cavarero (2000) points out, that “autobiographical memory always recounts a story that is incomplete from the beginning. It is necessary to go back to the narration told by others, in order for the story to begin from where it really began” (p. 39). The stories the adult Janet tells about herself and her father have their roots in her earliest experiences at home in the family, with those who knew her best. Yet even here, as she comes to realize, the status of her self-knowledge is precarious. She remembers the words of her father: “You know those years you were growing up—well, that’s all just a kind of a blur to me” (p. 222). In turn, she reflects on herself as a young parent who “couldn’t tell the years apart” (p. 223), and thinks regretfully about her exhaustion and forgetfulness at the time her daughters were born. It seems as if the first chapter in the story of a life is inescapably registered as a loss, even by those we have most trusted to keep, sustain, and treasure it. The quest for self-knowledge, for the self’s story of itself, can therefore only ever begin with a fiction, “a refabulation,” says Cavarero (2000), “of a story told by others” (p. 39). Indeed, we might recall that the poem by Joaquin Miller that Janet’s father tries to remember is itself a refabulation. It is a tale told by Columbus’s shipmate about the discovery of America, as well as the retelling of a founding story of origins. Munro’s insertion of Miller’s poem stages this retelling by offering us the story of a tale wrapped within a fable that is itself contained within a fiction. In “The Moons of Jupiter,” just as in *Totem and Taboo*, “realism [gives] way to familiar artifice,” offering the reader an exemplary tale about the death of the father that founds a New World, with both Munro and Freud thereby each creating a metafictional world whose exposition incarnates a constitutive truth about the formation of the subject.

The dizzyingly recursive fictions through which subjectivity is produced are one reason, Butler (2005) argues, that the self can never be reducible to narrative competence. When we try, like Janet, to give an account of ourselves, our narrative, she suggests, “begins *in media res*, when many things have already taken place to make me and my story possible in language. I am always recuperating, reconstructing, and I am left to fictionalize and fabulate origins I cannot know” (p. 37). Just as the *mise en abyme* of the planetarium scene in Munro’s tale offers an endless self-mirroring that blurs any putative distinction between fiction and reality, between model and what we can know of the real, so too we might say there is a “blur” at the heart of the subject: no single, distinct version or “ur-text” of the self but rather a variety of endlessly multiplying forms or narratives in all their “innumerable variations, innumerable repetitions.” It is this never ending deferral of meaning that destabilizes any foundational account of the self, opening up to us the “familiar artifice” of a subject whose narrative reconstruction is constantly undergoing revision. Indeed, Munro goes further by insisting we doubt the authenticity of any story we may come to tell about ourselves. For if my origin lies only in something I have fictioned, how can I affirm its truth? And insofar as my story is “dazzling, apt and striking,” can it ever be anything more than “an evasion”? By what means is it possible to have any kind of authoritative knowledge about the self?

## Conclusion

Authoritative knowledge, of course, tends to be knowledge whose reference point lies “out there” in the external world. Rooted in the everyday world of “morals and manners” (Martineau 1838), we might, if with some oversimplification, regard the novel as anchored in an external reference point that acts to bind the form, providing a basis for its moral and aesthetic integrity. The referential basis for the short story, however, is somewhat different. While the pith and brevity of Munro’s tale do not preclude reference to the external world, the story’s “self-consciousness and stylized arrangement” (McIntyre 2009, p. 74), its highly choreographed use of flashback, and above all its metafictional treatment of writing and representation, signal a refusal of contextual details and explanatory logic that would otherwise embed it within the solidarity of the social world. In what then is the short story anchored? Where is the source of its integrity? For the *mise en abyme* at the heart of Munro’s tale is not merely a clever postmodern literary device to demonstrate the self’s endless refractions of itself; rather, it contains folded within itself an implicit question about what makes for integrity when the very foundation of the subject cannot be guaranteed. How can the self, which represents most nearly “who I am,” be sustained in the short story’s disjointed and fragmentary way of telling? Recall that *The Moons of Jupiter* comprises eleven stories, each offering an example or *paradeigma* of women’s lives. But examples, as Keenan argues, are largely a matter of identification and substitution; and so by offering the reader a series of examples to identify with, we could say Munro’s final tale mimes how subjectivity is produced in the place where previously there was nothing but the event of a comparison. Indeed, her extraordinary title story is one that most clearly stands out as a resplendent exemplar of the self. Glittering against the cosmic backdrop of ten other fictive lives, “The Moons of Jupiter” dramatizes how subjectivity emerges, as it does in Aesop’s raven, by way of response to an alterity that always precedes it. In this way, the integrity of the self, like the integrity of the short story, can be thought of as sustained and authorized only by the fabulous artifice of its own origins.

What might this mean for us as psychoanalytic practitioners? In taking the short story as literary model for the subject, it seems we are faced with the responsibility of hearing an account of a life for which no other may be taken as a reference or model. We are exposed to the self’s irreducible singularity, in which no external frame of reference can be invoked by way of response. In our attempt to establish general laws, lessons, or rules that confer on a particular “case” what Freud (1895) liked to call “the serious stamp of science” (p. 160). We are apt to be misled by our use of limiting terms and definitions: “questionable tricks,” as Munro might say, that offer false comfort and reassurance in the face of the unknown. Indeed, just as a full view of all the moons of Jupiter is forever unavailable to us, so too our perspective on other people can only ever be fragmented and partial. “I ask my mind a question,” says Janet’s father, “but I can’t see all the connections my mind’s making to get it” (p. 225). In seeking, like Janet’s father, to “see all the connections” within the inner world of the mind, as well as in the external world of the cosmos, we reach out toward, yet inevitably fall short of, establishing the whole truth.

So we might consider to what extent Munro’s “way of telling” in the short story could be said to shine a light on the kind of insight that psychoanalytic work aims to foster. After all, in contemporary psychoanalysis the analyst, unlike the author who takes responsibility for creating a short story from his or her imagination, comes to play an extremely important role in co-constructing stories with the patient, a joint hermeneutic enterprise intended to persuade us of the meaning of a life (Mulligan 2017). Indeed, current analytic preoccupations with issues of intersubjectivity (see, e.g., Benjamin 1990; Ogden 1994, 2004) have familiarized us with the idea that such self-narratives come to form in part the patient’s constitutive identifications, not via a process of imposed narrative authority but via shifting interpersonal dynamics that sponsor, for example, moments of longed-for mutual recognition (Benjamin 2004). Reading the penultimate scene where Janet and her father work together to remember the names and stories associated with the moons of Jupiter, we are aware that this mutual effort, so close to the narrative work of analyst and patient, constitutes a final, implicit endeavor on the part of both to make meaningful emotional contact: a shared, fleeting attempt that is all the more powerful and touching for its brevity, restraint, and indirection. Janet and her father’s explanatory fictions here not only generate a comforting narrative that brings them together before their final parting at the hospital; it strikingly indexes the way in which language can paradoxically be a source of both communication and alienation, and how it can be used to at once both convey and camouflage emotion.^1^

So for all its “dazzling,” or untrustworthy nature, and despite the way it constantly “turn[s] out to be a questionable trick,” Munro wants to remind us that we cannot dispense with fiction. Like Winnicott’s capacity for illusion, recently discussed by Seligman (2018), it is crucial to, and indeed constitutes, our sense of reality. For in the “phony temple” of the fictioning self, it seems there is no objective, universal truth to which we can turn. All we have is the “moment that’s explosive,” the vivid “flash of fireflies” that constitutes a condensed, discrete *moment of truth*. “Any life,” says Borges (1968), “no matter how long or complex it may be, is made up essentially of a single moment” (p. 83). The short story’s way of telling is one that reveals to us that it is only the integrity of the moment itself—“the fleeting, emerging, and vanishing mental content” (Cassirer 1946, p. 18)—that can ultimately assume exemplary or representative status within a life. It is a moment to which we might well attend. For in the patient’s own “work with words”—within the telling of a dream, the temporary forgetfulness, the careless slip of the tongue—we may find, along with Munro and Freud, where the foundational fiction of the self, the subject, most unavoidably lies.

## Footnotes

1 While Munro’s moral struggle with language seems to focus mainly on its insufficiency in adequately representing reality, Winnicott (1963) might take a very different perspective, arguing that language risks exposing too much. “At the centre of each person is an incommunicado element,” he writes, “and this is sacred and most worthy of preservation. . . . traumatic experiences that lead to the organization of primitive defences belong to the threat to the isolated core, the threat of its being found, altered, communicated with. . . . Rape, and being eaten by cannibals, these are mere bagatelles as compared with the violation of the self’s core” (p. 187). Earlier in the story, Munro does not tell us why Janet’s adult daughter wishes to “remain incommunicado for a while” (p. 221), but we learn that in the past Janet had needed to withdraw emotionally when Nichola was a toddler and at risk of a diagnosis of leukemia: “I worried that she would feel the wind between the cracks of the manufactured holidays, the manufactured normal days” (p. 230). The connection between these events and Nichola’s subsequent refusal to communicate with Janet in adulthood is unarticulated by Munro, though it might be thought to imply that conversation between them risks exposing something traumatic in their relationship. Munro’s concern with “dramatizing what ought to be a most secret activity” (p. 217) thus seems to point to the potential for a double danger associated with the use of language: that it can be both too distant from reality and too revealing. The naming of the moons could be seen as pointing to a similar anxiety on the part of Janet and her father that speaking directly to each other about their relationship might be felt as too exposing.
